# Who Can Be Discharged Home after Adult Spinal Deformity Surgery?

**DOI:** 10.3390/jcm13051340

**Published:** 2024-02-27

**Authors:** Soren Jonzzon, Hani Chanbour, Graham W. Johnson, Jeffrey W. Chen, Tyler Metcalf, Alexander T. Lyons, Iyan Younus, Campbell Liles, Amir M. Abtahi, Byron F. Stephens, Scott L. Zuckerman

**Affiliations:** 1 Department of Neurological Surgery, Vanderbilt University Medical Center, Nashville, TN 37212, USA; soren.jonzzon@vumc.org (S.J.); hani.chanbour@vumc.org (H.C.); iyan.younus@vumc.org (I.Y.); david.c.liles.1@vumc.org (C.L.); amir.m.abtahi@vumc.org (A.M.A.); byron.stephens@vumc.org (B.F.S.); 2 School of Medicine, Vanderbilt University, Nashville, TN 37235, USA; graham.w.johnson@vanderbilt.edu (G.W.J.); alexander.t.lyons@vanderbilt.edu (A.T.L.); 3 Department of Neurological Surgery, Baylor College of Medicine, Houston, TX 77030, USA; jeffrey.chen@bcm.com; 4 Department of Orthopedic Surgery, Vanderbilt University Medical Center, Nashville, TN 37212, USA; metc11@osumc.edu

**Keywords:** adult spinal deformity, discharge disposition, home, SNF, IPR, scoring system

## Abstract

**Introduction:** After adult spinal deformity (ASD) surgery, patients often require postoperative rehabilitation at an inpatient rehabilitation (IPR) center or a skilled nursing facility (SNF). However, home discharge is often preferred by patients and hsas been shown to decrease costs. In a cohort of patients undergoing ASD surgery, we sought to (1) report the incidence of discharge to home, (2) determine the factors significantly associated with discharge to home in the form of a simple scoring system, and (3) evaluate the impact of discharge disposition on patient-reported outcome measures (PROMs). **Methods:** A single-institution, retrospective cohort study was undertaken for patients undergoing ASD surgery from 2009 to 2021. Inclusion criteria were ≥ 5-level fusion, sagittal/coronal deformity, and at least 2-year follow-up. Exposure variables included preoperative, perioperative, and radiographic data. The primary outcome was discharge status (dichotomized as home vs. IPR/SNF). Secondary outcomes included PROMs, such as the numeric rating scales (NRSs) for back and leg pain, the Oswestry Disability Index (ODI), and EQ-5D. A subanalysis comparing IPR to SNF discharge was conducted. Univariate analysis was performed. **Results:** Of 221 patients undergoing ASD surgery with a mean age of 63.6 ± 17.6, 112 (50.6%) were discharged home, 71 (32.2%) were discharged to an IPR center, and 38 (17.2%) were discharged to an SNF. Patients discharged home were significantly younger (55.7 ± 20.1 vs. 71.8 ± 9.1, *p* < 0.001), had lower rate of 2+ comorbidities (38.4% vs. 45.0%, *p* = 0.001), and had less hypertension (57.1% vs. 75.2%, *p* = 0.005). Perioperatively, patients who were discharged home had significantly fewer levels instrumented (10.0 ± 3.0 vs. 11.0 ± 3.4 levels, *p* = 0.030), shorter operative times (381.4 ± 139.9 vs. 461.6 ± 149.8 mins, *p* < 0.001), less blood loss (1101.0 ± 977.8 vs. 1739.7 ± 1332.9 mL, *p* < 0.001), and shorter length of stay (5.4 ± 2.8 vs. 9.3 ± 13.9 days, *p* < 0.001). Radiographically, preoperative SVA (9.1 ± 6.5 vs. 5.2 ± 6.8 cm, *p* < 0.001), PT (27.5 ± 11.1° vs. 23.4 ± 10.8°, *p* = 0.031), and T1PA (28.9 ± 12.7° vs. 21.6 ± 13.6°, *p* < 0.001) were significantly higher in patients who were discharged to an IPR center/SNF. Additionally, the operating surgeon also significantly influenced the disposition status (*p* < 0.001). A scoring system of the listed factors was proposed and was validated using univariate logistic regression (OR = 1.55, 95%CI = 1.34–1.78, *p* < 0.001) and ROC analysis, which revealed a cutoff value of > 6 points as a predictor of non-home discharge (AUC = 0.75, 95%CI = 0.68–0.80, *p* < 0.001, sensitivity = 63.3%, specificity = 74.1%). The factors in the scoring system were age > 56, comorbidities ≥ 2, hypertension, TIL ≥ 10, operative time > 357 mins, EBL > 1200 mL, preop SVA > 6.6 cm, preop PT > 33.6°, and preop T1PA > 15°. When comparing IPR (*n* = 71) vs. SNF (*n* = 38), patients discharged to an SNF were significantly older (74.4 ± 8.6 vs. 70.4 ± 9.1, *p* = 0.029) and were more likely to be female (89.5% vs. 70.4%, *p* = 0.024). **Conclusions:** Approximately 50% of patients were discharged home after ASD surgery. A simple scoring system based on age > 56, comorbidities ≥ 2, hypertension, total instrumented levels ≥ 10, operative time > 357 mins, EBL > 1200 mL, preop SVA > 6.6 cm, preop PT > 33.6°, and preop T1PA > 15° was proposed to predict non-home discharge. These findings may help guide postoperative expectations and resource allocation after ASD surgery.

## 1. Introduction

Symptomatic adult spinal deformity (ASD) currently affects between 2% and 32% of adults over 65 years old, and its prevalence continues to rise in North America as our population ages [1]. In recent years, surgery for the correction of ASD has grown in volume and complexity [2]. Though improvement in the quality of life is considerable [3], these operations are associated with high complication rates and long recovery periods [4,5,6]. As a result, patients often require post-acute care through inpatient rehabilitation (IPR) centers or skilled nursing facilities (SNFs) [7,8,9], rather than a direct discharge to home. 

Prior studies estimate that, following ASD surgery, 25% of patients are not discharged home [10]. Demographic factors associated with non-home discharge included age over 65, lower preoperative functional status, female sex, and obesity [7,8,10]. Additionally, longer operative time, three-column osteotomy, blood transfusion, and perioperative complications have been shown to increase the likelihood of non-home discharge [7,8,11]. Discharge to IPR centers/SNFs also increases patient and societal costs [7,9,12]. Moreover, the chance for facility-acquired infections may be higher at an IPR center/SNF than at home [13]. Understanding the factors that influence whether a patient can be discharged home or to an IPR center/SNF is crucial for patient expectations, postoperative management teams, and cost savings.

While existing research has shown various demographic and perioperative factors associated with non-home discharge, scarce research exists regarding the predictors of discharge to an IPR center/SNF with an easily implementable scoring system. In patients undergoing ASD surgery, we sought to (1) report the incidence of discharge to home, (2) determine the factors significantly associated with discharge to home in the form of a simple scoring system, and (3) evaluate the impact of discharge disposition on patient-reported outcome measures (PROMs).

## 2. Materials and Methods

### 2.1. Study Design

A single-institution, retrospective case–control study was queried from an ASD registry for patients from 2009 to 2021. Full-time employees completed and collected preoperative and postoperative PROMs. All cases within this registry involved an operation by one of five fellowship-trained neurosurgery and orthopedic spine surgeons. Institutional review board (IRB) approval was obtained (IRB# 220894).

### 2.2. Patient Selection

All patients within this study were ≥18 years old and underwent elective ASD surgery. The criteria for ASD surgery inclusion were ≥5-level fusions, Cobb angle ≥ 30°, sagittal vertical axis (SVA) ≥ 5 cm, coronal vertical axis (CVA) ≥ 3 cm, pelvic tilt (PT) ≥ 25°, or thoracic kyphosis (TK) ≥ 60°. Only patients with a minimum of two-year follow-up were included. Patients were included only if they had a clearly documented discharge disposition.

### 2.3. Independent Variables

Demographic and preoperative clinical data were collected, including age, sex, body mass index (BMI), smoking status, and the number of comorbidities (none, one, or two or more). Intraoperative data were gathered, including total instrumented levels (TILs), location of the upper instrumented vertebra (UIV), the number of interbody fusions, primary surgeon, and blood loss as measured by estimated blood loss (EBL). Postoperatively, perioperative hemoglobin, discharge disposition, and length of stay (LOS) were collected. 

Preoperative and postoperative radiographic measures were measured, including coronal/sagittal alignment. Coronal alignment was represented by the CVA, defined as the distance between the C7 plumb line (C7PL) and the central sacral vertical line (CSVL), and the major Cobb angle [14]. Sagittal measurements included the sagittal vertical axis (SVA) [14], defined as the distance between the C7 plumb line and the posterior–superior corner of S1, as well as the lumbar lordosis (LL), pelvic incidence (PI), sacral slope (SS), PT, T1 pelvic angle (TPA), and L1-S1 angle. Sagittal alignment was also classified using the Roussouly classification [15].

Postoperative two-year PROMs included the Oswestry Disability Index (ODI) [16], EuroQoL Group (EQ-5D) [17], and numeric rating scales (NRSs) for back and leg pain (NRSBP and NRS-LP, respectively). 

### 2.4. Outcome Variables

The primary outcome was discharge disposition, collected as home, IPR, or SNF, which was further binarized into home vs. non-home discharge. Discharge disposition was a multidisciplinary decision made by our physical therapy (PT) and occupational therapy (OT) colleagues, the surgical team, the patient, and their family. Though patients were often discharged in keeping with PT/OT recommendations, there were select scenarios where the patient and family wishes differed from the ascribed disposition. For example, if a patient was ambulating well enough to be discharged but lived alone, they may choose to be discharged to an IPR center or an SNF. Conversely, if a patient is not quite steady enough for home, but they have a lot of family support, they may opt for home discharge. Among those with non-home discharge, patients were further categorized into IPR or SNF, which was determined by our physical therapy and occupational therapy colleagues. 

### 2.5. Statistical Analysis 

Descriptive statistics were determined comparing ASD patients based on the discharge disposition. Continuous variables were reported as means and standard deviations, while categorical variables were reported as frequencies. Normal distribution and variance for continuous variables were assessed using the Shapiro–Wilk test and F-test, respectively. Qualitatively, histograms assisted in assessing for normality. A two-sample *t*-test was used for normally distributed data with equal variance, and the Wilcoxon signed-rank test or Mann–Whitney test was used for nonparametric data. Chi-squared or Fisher’s exact test in smaller samples was used for comparing nominal data. A primary analysis was completed for patients who underwent home vs. non-home discharge. Univariate logistic regression was performed, along with the receiver operator characteristic (ROC) curve for continuous variables, to calculate the area under the curve (AUC), Youden’s Index, cutoff values, sensitivity, and specificity. From the univariate analysis, a simple scoring system was proposed to better predict discharge to home. A subanalysis was subsequently performed for patients who were discharged to an SNF vs. IPR. A significance level was considered statistically significant when the alpha value was <0.05. All analyses were performed using R version 4.2.1 (The R Foundation, Vienna, Austria). 

## 3. Results

### 3.1. Patient Demographics

**Figure 1:**
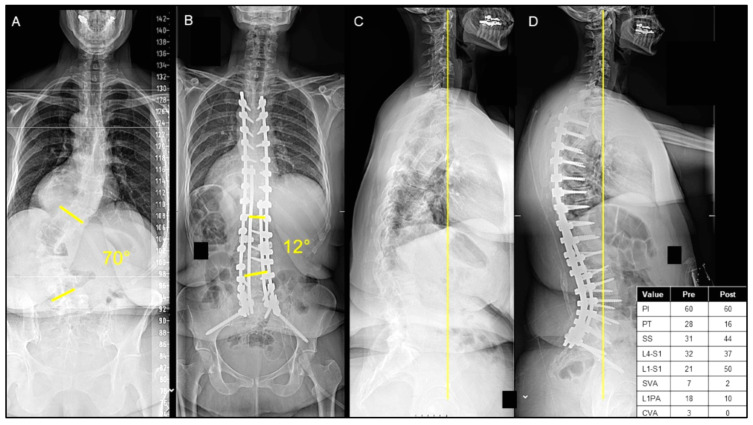
(**A**–**D**) A case presentation of a 49-year-old female presenting with adult idiopathic scoliosis and significant back pain with a significant sagittal/coronal malalignment, as seen on posterior–anterior (PA) (**A**) and lateral X-rays (**C**). The patient underwent T4 pelvis posterior spinal instrumentation with T11-L5 posterior column osteotomies, as seen on the postoperative PA (**B**) and lateral X-rays (**D**). The patient was discharged home on postoperative day 13.

**Figure 2:**
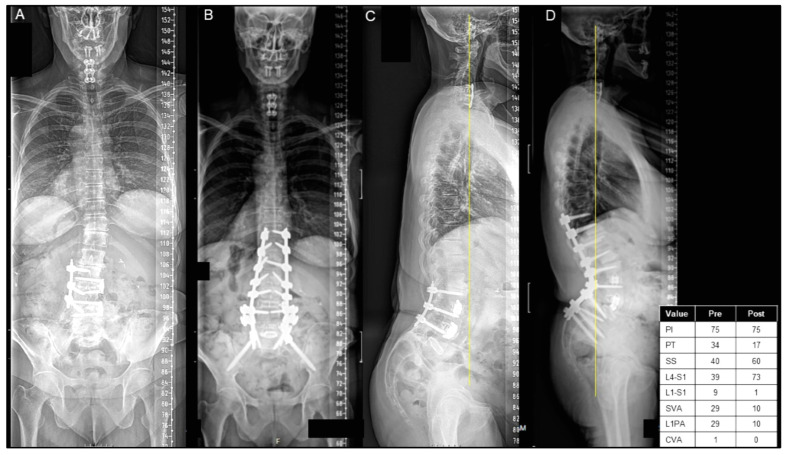
(**A**–**D**) A case presentation of a 61-year-old female with a history of an L5/S1 high-grade spondylolisthesis in situ fusion as a teenager and an L3–5 lateral fusion as an adult, presenting with back and leg pain and significant sagittal/coronal malalignment, as seen on posterior–anterior (PA) (**A**) and lateral X-rays (**C**). The patient underwent T10 pelvis posterior spinal instrumentation with an L4 PSO, as seen on postoperative PA (**B**) and lateral X-rays (**D**). The patient was discharged to IPR on postoperative day 7.

Of the 221 patients undergoing ASD surgery with a mean age of 63.6 ± 17.6 years, 112 (50.6%) were discharged home, 71 (32.2%) were discharged to an IPR center, and 38 (17.2%) were discharged to an SNF. Patients discharged home were significantly younger (55.7 ± 20.1 vs. 71.8 ± 9.1, *p* < 0.001) and less commonly had 2+ comorbidities (38.4% vs. 45.0%, *p* = 0.001), with no significant difference in sex (*p* = 0.609) (Table 1). Representative cases are shown of a patient who was discharged home after ASD surgery (Figure 1A–D) and discharged to an IPR center (Figure 2A–D).

### 3.2. Intraoperative and Perioperative Factors

Intraoperatively, patients discharged home had fewer TILs (10.0 ± 3.0 vs. 11.0 ± 3.4, *p* = 0.030), shorter operative durations (381.4 ± 139.9 vs. 461.6 ± 149.8 mins, *p* < 0.001), less estimated blood loss (1101.0 ± 977.8 vs. 1739.7 ± 1332.9 mL, *p* < 0.001), a smaller decrease in hemoglobin postoperatively (4.2 ± 2.5 vs. 5.2 ± 2.9 g/dL, *p* = 0.014), and shorter overall LOS (5.4 ± 2.8 vs. 9.3 ± 13.9 days, *p* < 0.001) compared to patients with non-home discharge disposition. The operating surgeon also significantly impacted patient disposition (*p* < 0.001). There were 15 surgeons, and their discharge-to-home rates were 25.0%, 14.3%, 12.5%, 10.7%, and others (37.5%). No significant difference was observed in the presence of interbody grafts at any level between the two cohorts (30.4% vs. 25.7%, *p* = 0.127) (Table 1) 

### 3.3. Radiographic Factors

Preoperative radiographic measures did not show significant differences in the CVA (2.5 ± 2.6 vs. 27.1 ± 26.4 cm, *p* = 0.574), sagittal L1–L4 (3.2 ± 18.4° vs. 8.2 ± 18.1°, *p* = 0.060), lumbar lordosis (19.5 ± 30.7° vs. 25.1 ± 35.3°, *p* = 0.072), PI (52.4 ± 14.8° vs. 52.9 ± 15.9°, *p* = 0.920), or SS (25.0 ± 12.1° vs. 29.7 ± 13.4°, *p* = 0.021). However, the SVA (9.1 ± 6.5 vs. 5.2 ± 6.8 cm, *p* < 0.001), PT (27.5 ± 11.1° vs. 23.4 ± 10.8°, *p* = 0.031), and T1 PA (28.9 ± 12.7° vs. 21.6 ± 13.6°, *p* < 0.001) were significantly higher in patients who were not discharged home, and the major coronal Cobb angle was significantly higher in patients who were discharged home (33.5 ± 16.8° vs. 25.8 ± 14.1°, *p* < 0.001).

Postoperative radiographic correction findings, including the CVA (3.2 ± 3.38 vs. 2.8 ± 2.7 cm, *p* = 0.640), major Cobb angle (32.8 ± 26.1° vs. 26.0 ± 21.3°, *p* = 0.163), SVA (4.6 ± 5.2 vs. 4.7 ± 3.9 cm, *p* = 0.928), T1PA (9.6 ± 9.0° vs. 10.0 ± 7.6°, *p* = 0.361), PT (7.2 ± 6.3° vs. 7.4 ± 7.4°, *p* = 0.976), and PI (9.9 ± 10.4° vs. 10.6 ± 11.4°, *p* = 0.427), did not show significant differences regarding home vs. non-home discharge. 

### 3.4. Scoring System

After univariate logistic regression, older age, more comorbidities, hypertension, more TILs, longer operative time, more EBL, higher preoperative SVA, higher preoperative PT, and higher preoperative T1PA were all predictors of non-home discharge (Table 2). The area under the curve and Youden’s Index for the continuous variables are summarized in Table 3.

Using the odds ratios found in the logistic regression analysis and the cutoff values calculated in the ROC analysis, the following scoring system (total = 11 points) was proposed: age > 56 years, one point; comorbidities (2+), one point; hypertension, two points; TIL > 10 levels, one point; operative time > 357 mins, one point; EBL > 1200 mL, one point; preop SVA > 6.6 cm, one point; preop PT > 33.6°, one point; and preop T1PA > 15°, one point.

**Figure 3:**
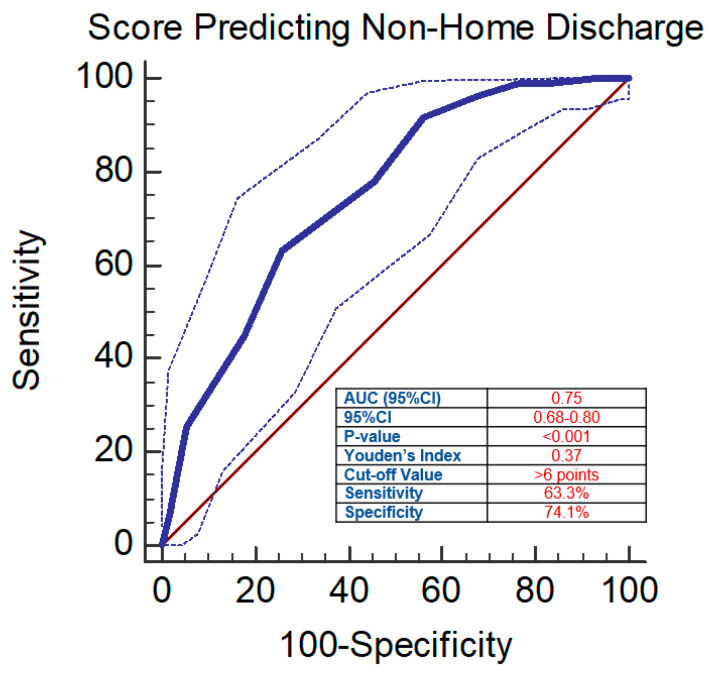
ROC curve and Youden’s Index of the scoring system predicting non-home discharge.

To validate this score, univariate logistic regression revealed that a higher score was associated with significantly higher odds of non-home discharge (OR = 1.55, 95%CI = 1.34–1.78, *p* < 0.001). ROC analysis showed a moderately high Youden’s Index of 0.37, with a cutoff value of > 6 points as a predictor of non-home discharge (AUC = 0.75, 95%CI = 0.68–0.80, *p* < 0.001, sensitivity = 63.3%, specificity = 74.1%) (Figure 3, Table 4).

### 3.5. Patient-Reported Outcome Measures

At 2 years postoperatively, no difference was found in any of the PROMs, namely the ODI (*p* = 0.158), NRS—Back pain (*p* = 0.459), NRS—Leg pain (*p* = 0.092), and EQ-5D (*p* = 0.134), between patients with home vs. non-home discharge (Table 1). 

### 3.6. Subanalysis of SNF vs. IPR

A total of 109 patients were discharged to an SNF (*n* = 38, 34.9%) or an IPR center (*n* = 71, 65.1%). Patients discharged to an SNF were significantly older (74.4 ± 8.6 vs. 70.4 ± 9.1, *p* = 0.029) and were mostly females (89.5% vs. 70.4%, *p* = 0.024), as summarized in Table 5. 

Intraoperatively, patients did not show significant differences in total instrumented levels (*p* = 0.761), the number of interbody grafts placed (*p* = 0.977), operative duration (*p* = 0.954), blood loss (*p* = 0.661), or LOS (*p* = 0.664). Similarly, no difference was found regarding preoperative or postoperative radiographic measurements, or postoperative PROMs, except a higher EQ-5D in patients discharged to SNFs (0.8 ± 0.1 vs. 0.6 ± 0.2, *p* = 0.011).

## 4. Discussion

The current study investigated the risk factors associated with non-home discharge disposition after ASD surgery. Patients with non-home discharge were older and had more comorbidities. Intraoperatively, patients undergoing complex surgeries with more TILs and more blood loss were more likely not to be discharged home. Radiographically, preoperative SVA, PT, and T1PA were higher in patients with non-home discharge. Based on these factors, a simple scoring system was proposed to predict non-home discharge. Comparing SNF vs. IPR disposition, older patients and females were significantly more often discharged to SNFs. Importantly, at 2 years postoperatively, no difference was found in any of the PROMs between those discharged to home vs. to an IPR center/SNF. The current study shows that this simple scoring can be used to allocate resources, set patient expectations, and assist in the determination of appropriate discharge disposition after ASD surgery. 

The current study reported a similar rate of home discharge compared to the prior literature with a reported range of 58–76% home discharge, with the highest rate of home discharge noted by Di Capua et al. [7,8,10]. In our cohort, fewer levels of instrumentation, less blood loss, and shorter operative time were all predictive of home discharge, although no difference was found in intraoperative and perioperative factors between SNF vs. IPR disposition. All of these variables are suggestive of a less complex procedure, which portends an improved postoperative course and increased likelihood of going home upon discharge, which is consistent with previous research [7,10,11]. In a study involving 1978 patients undergoing ASD surgery, Passias et al. [7] found similar predictors of non-home discharge, including age, female sex, and length of stay. In contrast, we did not find that the number of interbodies was a predictor of non-home discharge. Though complex surgeries may be the reason for a longer LOS in our study, discharge to an IPR center/SNF may be negatively impacted by delays in discharge awaiting bed availability. Furthermore, our data revealed that the surgeon involved had a statistically significant impact on the discharge disposition, which further emphasizes the multifactorial nature of the discharge process. Other surgeon-specific factors playing a role in the discharge disposition include the surgical complexity of the surgery and postoperative recovery preferences. 

There was no difference in the majority of preoperative and postoperative radiographic variables. However, a higher preoperative SVA, PT, and T1PA found in patients with non-home discharge was consistent with the results of Eastlack et al. [11], who also found that preoperative PT was associated with non-home discharge. Notably, the differences in PT and T1PA were 4.1° and 7.3°, respectively. While these values represent a relatively small discrepancy in preoperative variables, it may suggest that individuals with a higher PT and T1PA may have a more challenging postoperative course with regard to the correction of their deformity or adjustments to their deformity correction, which necessitates a higher level of post-acute care. Additionally, patients with a higher SVA often require larger correction to achieve postoperative goals, suggesting a larger deformity correction. While some variables have been previously reported in the literature, our study integrated various risk factors into a simple and practical scoring system, offering spine surgeons and patients a valuable tool to identify patients at higher risk of non-home discharge following ASD surgery. The scoring system facilitates more informed decision making regarding postoperative care and discharge planning, enhancing the clinical utility of these risk factors and improving patient outcomes.

With regard to 2-year PROMs, no difference was found between patients who were discharged home and those discharged to a non-home facility, namely an SNF or an IPR center. Although patients may have higher postoperative care needs that require post-acute care, their recovery at the 2-year time point is not limited by these postoperative needs or by their time in post-acute care. Despite the similarities with the study findings of Eastlack et al. [11], Amin et al. [8] found worse PROMs in patients with non-home discharge. 

Beyond the immediate postoperative period, factors such as access to follow-up care, social support, socioeconomic status, financial burden, and rehabilitation services may have a considerable impact on non-home discharge. For instance, the availability of strong social support networks can facilitate smoother transitions from hospital to home, reducing the likelihood of non-home discharge. Moreover, disparities in access to rehabilitation services, such as physical therapy or home healthcare, may disproportionately affect certain patient populations with more or less economic resources, contributing to variations in discharge disposition. Recognizing the interplay between these factors and discharge disposition is crucial for optimizing patient care and mitigating the barriers to successful discharge to home. Notably, the applicability of the predictor formula may be limited by regional-specific factors such as local policies and the medical insurance systems of the United States.

The current study is not without limitations. First, the study is retrospective in nature, which has its own inherent biases regarding interpretability and causality. Second, this study represents the findings of a single institution and would need to be externally validated using larger, multicenter prospective studies. Despite the valuable insights gained from our single-institution study, it is essential to recognize the potential limitations associated with the exclusive focus on one medical center and the relatively small sample size. The unique patient population and surgical practices at our institution may limit the generalizability of the findings to a broader spectrum of patients undergoing ASD surgery. Future studies involving multiple institutions and larger cohorts are warranted to validate and extend our observations to a more diverse patient population. Third, multivariable regression including all the significant factors of bivariate analysis overwhelmed the regression model due to the limited sample size, which subsequently prompted us to only pursue univariate analysis. The scoring system was derived from univariate analysis only, which certainly weakens the results and limits the power of the proposed scoring system. Findings such as why hypertension was associated with non-home discharge are correlational, and we were not able to assess whether a causal relationship exists between hypertension and non-home discharge. It is also possible that hypertension is a surrogate for overall health status, and patients with more comorbidities were more likely to have a non-home discharge. Lastly, our data revealed that the surgeon involved in the case had a statistically significant impact on the discharge disposition, which further emphasizes the multifactorial nature of disposition and the careful interpretation of our findings. Surgeon preference could alter multiple factors such as surgical approach, surgical technique, or postoperative recovery preferences, although all surgeons should have similar goals, and our scoring system aims to be applicable to all surgeons. Moreover, retrospective studies inherently carry the risk of selection bias, and it is essential to acknowledge this limitation. The retrospective nature of our study means that patient selection may have been influenced by factors beyond our control, potentially introducing bias into our findings. Factors such as access to healthcare, socioeconomic status, and patient preferences could influence who undergoes surgery and subsequently impact discharge disposition. Therefore, a cautious interpretation of our results is warranted, recognizing the limitations imposed by the retrospective design. Moving forward, prospective studies could help control for these factors more effectively, providing a clearer understanding of the relationship between patient characteristics, surgical outcomes, and discharge disposition in the context of ASD surgery. 

## 5. Conclusions

In patients undergoing ASD surgery, we emphasized the multifaceted determinants of postoperative discharge disposition. We proposed a scoring system including age > 56, comorbidities ≥ 2, hypertension, TIL ≥ 10, operative time > 357 mins, EBL > 1200 mL, preop SVA > 6.6 cm, preop PT > 33.6°, and preop T1PA > 15° to predict non-home discharge. We also found that discharge disposition did not impact 2-year PROMs. Comparing SNF vs. IPR, older age and female gender were associated with an increased likelihood of discharge to an SNF. These findings underscore the importance of postoperative care plans tailored to specific demographic characteristics to address patients’ needs and involve them in the decision-making process.

## Figures and Tables

**Table 1:** Comparing preoperative, intraoperative, and postoperative variables between patients with home vs. non-home discharge.

Variables		Total Cohort *n* = 221	Home Discharge *n* = 112	Non-Home Discharge *n* = 109	*p*-Value
**Preoperative**					
Age, mean ± SD		63.6 ± 17.6	55.7 ± 20.1	71.8 ± 9.1	<0.001
Female, *n* (%)		167 (75.6%)	83 (74.1%)	84 (77.1%)	0.262
BMI, mean ± SD		28.9 ± 7.0	28.3 ± 7.6	29.4 ± 6.3	0.088
Comorbidities, *n* (%)	0	44 (19.9%)	33 (29.5%)	11 (10.1%)	0.001
	1	85 (38.5%)	36 (32.1%)	49 (45.0%)	
	2+	92 (41.6%)	43 (38.4%)	49 (45.0%)	
Diabetes, *n* (%)		41 (18.6%)	17 (15.2%)	24 (22.0%)	0.191
COPD, *n* (%)		61 (27.6%)	31 (27.7%)	30 (27.5%)	0.979
CHF, *n* (%)		32 (14.5%)	13 (11.6%)	19 (17.4%)	0.219
HTN, *n* (%)		146 (66.1%)	64 (57.1%)	82 (75.2%)	0.005
Osteoporosis, *n* (%)		43 (24.7%)	17 (18.9%)	26 (31.0%)	0.065
Prior fusion, *n* (%)		77 (34.8%)	35 (31.2%)	42 (38.5%)	0.256
**Intraoperative**					
Total instrumented levels, mean ± SD		10.5 ± 3.2	10.0 ± 3.0	11.0 ± 3.4	0.030
Interbody graft at any level, *n* (%)		62 (28.0%)	34 (30.4%)	28 (25.7%)	0.127
Operative time, min, mean ± SD		421.1 ± 150.0	381.4 ± 139.9	461.6 ± 149.8	<0.001
EBL, mL, mean ± SD		1416.0 ± 1207.0	1101.0 ± 977.8	1739.7 ± 1332.9	<0.001
LOS, days, mean ± SD		7.3 ± 10.1	5.4 ± 2.8	9.3 ± 13.9	<0.001
**2-year Postoperative**					
ODI, mean ± SD		35.2 ± 19.7	32.5 ± 22.3	37.1 ± 17.4	0.158
NRS-Back pain, mean ± SD		4.8 ± 2.9	4.6 ± 3.0	5.0 ± 2.9	0.459
NRS-Leg pain, mean ± SD		2.8 ± 3.3	2.2 ± 2.9	3.2 ± 3.4	0.092
EQ-5D, mean ± SD		0.7 ± 0.2	0.7 ± 0.2	0.7 ± 0.2	0.134

**Table 2:** Univariate logistic regression of the predictors of home vs. non-home discharge and SNF vs. IPR.

		Univariate	
Outcome Variable	Independent Variable	OR (95%CI)	*p*-Value
Non-home discharge	Age	1.10 (1.05–1.12)	<0.001
	Comorbidities (2+)	1.60 (1.11–2.28)	0.011
	HTN	2.30 (1.28–4.04)	0.005
	TIL	1.09 (1.005–1.19)	0.037
	Operative time, min	1.004 (1.002–1.01)	<0.001
	EBL	1.001 (1.00–1.001)	<0.001
	Preop SVA	1.01 (1.005–1.014)	<0.001
	Preop PT	1.03 (1.01–1.06)	0.007
	Preop T1PA	1.04 (1.02–1.06)	<0.001

**Table 3:** AUC values and Youden’s Index of the continuous variables as predictors of non-home discharge.

Predictors of Non-Home Discharge	AUC (95%CI)	Youden’s Index	Cutoff Value	*p*-Value
Age	0.75 (0.69–0.80)	0.36	>56 years	<0.001
TIL	0.58 (0.51–0.65)	0.13	>10 levels	0.027
Operative time, min	0.68 (0.62–0.74)	0.38	>357 min	<0.001
EBL	0.67 (0.61–0.73)	0.28	>1200 mL	<0.001
Preop SVA	0.66 (0.59–0.72)	0.28	>6.6 cm	<0.001
Preop PT	0.58 (0.51–0.65)	0.14	>33.6°	0.025
Preop T1PA	0.64 (0.58–0.71)	0.23	>15°	>0.001

**Table 4:** Scoring system predicting non-home discharge.

Predictors of Non-Home Discharge	Score (10 Points)
Age > 56 years	1
Comorbidities (2+)	1
Hypertension	2
TIL > 10 levels	1
Operative time, > 357 min	1
EBL > 1200 mL	1
Preop SVA > 6.6 cm	1
Preop PT > 33.6°	1
Preop T1PA > 15°	1
**Validation**	
Logistic regression	OR = 1.55, 95%CI = 1.34–1.78, *p* < 0.001
AUC (95%CI)	AUC = 0.75, 95%CI = 0.68–0.80, *p* < 0.001
Youden’s Index	0.37
Cut-off value	6 points
Score > 6	More likely to be discharged to IPR/SNF
Score ≤ 6	More likely to be discharged home
Sensitivity	63.3%
Specificity	74.1%

**Table 5:** Comparing preoperative, intraoperative, and postoperative variables between patients with IPR vs. SNF discharge.

Variables		Non-Home Discharge *n* = 109	IPR (*n* = 71)	SNF (*n* = 38)	*p*-Value
**Preoperative**					
Age, mean ± SD		71.8 ± 9.1	70.4 ± 9.1	74.4 ± 8.6	0.029
Female, *n* (%)		84 (77.1%)	50 (70.4%)	34 (89.5%)	0.024
BMI, mean ± SD		29.4 ± 6.3	28.9 ± 6.0	30.4 ± 6.8	0.283
Comorbidities, *n* (%)	0	11 (10.1%)	6 (8.5%)	5 (13.2%)	0.738
	1	49 (45.0%)	32 (45.1%)	17 (44.7%)	
	2+	49 (45.0%)	33 (46.5%)	16 (42.1%)	
Diabetes, *n* (%)		24 (22.0%)	16 (22.5%)	8 (21.1%)	0.859
COPD, *n* (%)		30 (27.5%)	20 (28.2%)	10 (26.3%)	0.836
CHF, *n* (%)		19 (17.4%)	14 (19.7%)	5 (13.2%)	0.39
HTN, *n* (%)		82 (75.2%)	53 (74.6%)	29 (76.3%)	0.848
Osteoporosis, *n* (%)		26 (31.0%)	16 (29.6%)	10 (33.3%)	0.725
Prior fusion, *n* (%)		42 (38.5%)	27 (38.0%)	15 (39.5%)	0.885
**Intraoperative**					
Total instrumented levels, mean ± SD		11.0 ± 3.4	10.9 ± 3.6	11.0 ± 2.9	0.761
Interbody graft at any level, *n* (%)		28 (25.7%)			
Operative time, min, mean ± SD		461.6 ± 149.8	465.7 ± 158.3	453.9 ± 134.3	0.954
EBL, mL, mean ± SD		1739.7 ± 1332.9	1658.2 ± 1226.9	1892.1 ± 1517.0	0.661
LOS, days, mean ± SD		5.4 ± 2.8	8.6 ± 8.5	10.5 ± 20.5	0.664
**2-year Postoperative**					
ODI, mean ± SD		37.1 ± 17.4	39.9 ± 18.3	32.2 ± 14.8	0.098
NRS—Back pain, mean ± SD		5.0 ± 2.9	5.4 ± 2.9	4.1 ± 2.8	0.065
NRS—Leg pain, mean ± SD		3.2 ± 3.4	3.7 ± 3.5	2.4 ± 3.2	0.158
EQ-5D, mean ± SD		0.7 ± 0.2	0.6 ± 0.2	0.8 ± 0.1	0.011

## Data Availability

For data inquiries, please contact Scott L. Zuckerman.

## References

[1] Cerpa M., Lenke L.G., Fehlings M.G., Shaffrey C.I., Cheung K.M.C., Carreon L.Y. (2019). Evolution and Advancement of Adult Spinal Deformity Research and Clinical Care: An Overview of the Scoli-RISK-1 Study. Glob. Spine J..

[2] Safaee M.M., Ames C.P., Smith J.S. (2020). Epidemiology and Socioeconomic Trends in Adult Spinal Deformity Care. Neurosurgery.

[3] Riley M.S., Bridwell K.H., Lenke L.G., Dalton J., Kelly M.P. (2018). Health-related quality of life outcomes in complex adult spinal deformity surgery. J. Neurosurg. Spine.

[4] Daubs M.D., Lenke L.G., Cheh G., Stobbs G., Bridwell K.H. (2007). Adult Spinal Deformity Surgery: Complications and Outcomes in Patients Over Age 60. Spine.

[5] Yoshida G., Boissiere L., Larrieu D., Bourghli A., Vital J.M., Gille O., Pointillart V., Challier V., Mariey R., Pellisé F. (2017). Advantages and Disadvantages of Adult Spinal Deformity Surgery and Its Impact on Health-Related Quality of Life. Spine.

[6] Scheer J.K., Mundis G.M., Klineberg E., Hart R.A., Deviren V., Nguyen S., Protopsaltis T.S., Gupta M., Bess S., Shaffrey C.I. (2015). Postoperative Recovery after Adult Spinal Deformity Surgery: Comparative Analysis of Age in 149 Patients during 2-year Follow-up. Spine.

[7] Passias P.G., Poorman G.W., Bortz C.A., Qureshi R., Diebo B.G., Paul J.C., Horn S.R., Segreto F.A., Pyne A., Jalai C.M. (2018). Predictors of adverse discharge disposition in adult spinal deformity and associated costs. Spine J..

[8] Amin R.M., Raad M., Jain A., Khashan M., Hassanzadeh H., Frank S.M., Kebaish K.M. (2019). Risk factors for nonroutine discharge in adult spinal deformity surgery. Spine J..

[9] Theologis A.A., Lau D., Dalle-Ore C., Tsu A., Deviren V., Ames C.P. (2021). Costs and utility of post-discharge acute inpatient rehabilitation following adult spinal deformity surgery. Spine Deform..

[10] Di Capua J., Somani S., Lugo-Fagundo N., Kim J.S., Phan K., Lee N.J., Kothari P., Shin J., Cho S.K. (2018). Predictors for Non-Home Patient Discharge Following Elective Adult Spinal Deformity Surgery. Glob. Spine J..

[11] Eastlack R.K., Ledesma J.B., Tran S., Khalsa A., Park P., Mummaneni P.V., Chou D., Kanter A.S., Anand N., Nunley P. (2018). Home Versus Rehabilitation: Factors that Influence Disposition After Minimally Invasive Surgery in Adult Spinal Deformity Surgery. World Neurosurg..

[12] Stephens B.F., Khan I., Chotai S., Sivaganesan A., Devin C.J. (2018). Drivers of Cost in Adult Thoracolumbar Spine Deformity Surgery. World Neurosurg..

[13] Montoya A., Mody L., Sanroma P., Muñoz P., Mirón-Rubio M., Aguilera A., Estrada O., García D., González-Ramallo V.J., Pajarón M. (2011). Common infections in nursing homes: A review of current issues and challenges. Aging Health.

[14] Lee C.S., Ha J.-K., Kim D.G., Kim H., Hwang C.J., Lee D.-H., Cho J.H. (2015). The clinical importance of sacral slanting in patients with adolescent idiopathic scoliosis undergoing surgery. Spine J..

[15] Roussouly P., Gollogly S., Berthonnaud E., Dimnet J. (2005). Classification of the normal variation in the sagittal alignment of the human lumbar spine and pelvis in the standing position. Spine.

[16] Fairbank J.C., Couper J., Davies J.B., O’Brien J.P. (1980). The Oswestry low back pain disability questionnaire. Physiotherapy.

[17] The EuroQol Group (1990). EuroQol—A new facility for the measurement of health-related quality of life. Health Policy.

